# The Story of the Insane from Year to Year

**Published:** 1894-09-08

**Authors:** 


### THE STORY OF THE INSANE FROM YEAR TO YEAR.

(SEVENTH SERIES.)

### NORTHUMBERLAND COUNTY ASYLUM.

On the last day of the year there were 603 patients in residence, showing an increase of 45 since January last. The admissions were 105, the recoveries 43, and the deaths 68. These numbers yield 29·9 per cent. of recoveries and 11·6 of deaths. Hereditary influence is the stated cause of insanity in 30 of the admissions, and drink in 21. Thirty-four of the deaths were due to some form of cerebral or spinal disease, and consumption was the cause in 13. There was one death from typhoid fever, and Dr. McDowall says "the disease must have originated from some cause or causes within the asylum, as the patient had not been out of it for at least twenty years; but most diligent search failed to discover it." Dysenteric diarrhœa was the cause of death in two cases, and no cause could be detected for its appearance; and this is precisely what has happened in many other asylums. Typhoid and dysentery have appeared at intervals where the water was above suspicion and the drainage excellent. The visitors say "they are perfectly satisfied with the efficient manner in which Dr. McDowall has performed the duties of his office." The weekly rate is 9s. 5⅝d.

### THE BERKSHIRE ASYLUM, MOULSFORD.

In Table I. of the medical statistics there is a misprint of "1893" for "1892"; but the meaning is plain enough, and the number of patients at the end of the year was the same as at the beginning, namely, 545. There were 106 admissions, 38 recoveries, and 49 deaths. The percentage of recoveries was 35·8, and that of the deaths was 9·0. Hereditary tendency alone, or in combination with other causes, was instrumental in causing the insanity in 34 of the year's admissions. Similarly intemperance in drink accounts for 8. Twenty-one of the deaths were owing to some form of brain disease, and 5 to consumption. Dysenteric diarrhœa was the cause in one instance. Dr. Murdoch must have had his hands full during the year, for we find he had 136 of the patients and 42 of the staff stricken with influenza; 7 patients and 5 of the staff had scarlatina, 3 had typhoid fever, and epidemics of ophthalmia and diarrhœa appeared in the autumn. The committee record their "great satisfaction at the able manner in which all the affairs of the asylum have been managed by Dr. Murdoch." The weekly charge to the unions is 8s. 2d. per head.

### SURREY COUNTY ASYLUM, BROOKWOOD.

Here the number decreased from 1,055 to 1,019. There were 278 admissions, 87 recoveries, and 155 deaths, and these numbers show that the percentage of recoveries on admissions was 31·33, and that of the deaths 14·83 on the average number resident. Intemperance in drink was the assigned cause of insanity in 37 of the admissions, and hereditary influence in 84. Religious excitement is credited with being the cause in 11, and in 39 there was no cause known. Cerebral and spinal diseases were the causes of death in 57 instances, and consumption in 6. Dr. Barton has not been behind other medical superintendents in the training of his staff of nurses and attendants. Thirty-six of them obtained the St. John Ambulance certificate and 12 passed the examination of the Medico-Psychological Association. We are glad to congratulate the farm bailiff on obtaining his pension of £60 per annum. The visitors "express their satisfaction with the management of the asylum and the conduct of the staff," and they say they have not felt justified in adopting the recommendation of the Commissioners in Lunacy relative to the appointment of an additional medical officer with a view to scientific research. However desirable such work may be, we have all along doubted whether the rates can be legally applied to such a purpose. The weekly charge to the unions is 9s. 6d.

